# Effects of Individual Changes in Training Distribution on Maximal Aerobic Capacity in Well-Trained Cross-Country Skiers: A Follow-Up Study

**DOI:** 10.3389/fphys.2021.675273

**Published:** 2021-06-28

**Authors:** Jan-Michael Johansen, Arnstein Sunde, Jan Helgerud, Lars Erik Gjerløw, Øyvind Støren

**Affiliations:** ^1^Department of Natural Sciences and Environmental Health, University of South-Eastern Norway, Bø, Norway; ^2^Department of Sports, Physical Education and Outdoor Studies, University of South-Eastern Norway, Bø, Norway; ^3^Department of Circulation and Medical Imaging, Norwegian University of Science and Technology, Trondheim, Norway; ^4^Myworkout, Medical Rehabilitation Centre, Trondheim, Norway

**Keywords:** endurance, VO_2max_, total training volume, cross country skiing, training characteristic

## Abstract

The purpose of this study was to evaluate individual changes in training distribution and the subsequent effects on maximal oxygen uptake (VO_2max_). The participants were well-trained cross-country skiers who had performed a year with no substantial changes in training prior to this study. Six cross-country skiers, who were participants in a larger previous study, volunteered for a follow-up study. All skiers performed self-motivated changes in training distribution for a new preparation period in this follow-up, generally by more high-intensity training (HIT). All training characteristics were registered from training diaries. During the follow-up period, all skiers performed an incremental VO_2max_ test in February 2020 and August 2020. Training were categorized into three different training periods; (1) February 2019 to February 2020 (*P*_1_) representing the training performed prior to the follow-up, (2) February 2020 to July 2020 (*P*_2_), and (3) July 2020 to August 2020 (*P*_3_). On average, the skiers increased their VO_2max_ by 5.8 ± 5.0% (range: −1.8 to + 10.2%) during the follow-up study compared with the average VO_2max_ during the preceding year. Total training volume increased on average by 10.0 and 25.7% in *P*_2_ and *P*_3_, respectively, compared with *P*_1_. The average volume of HIT was similar between *P*_1_ and *P*_2_ but increased 62.8% in *P*_3_. However, large individual differences in training changes were observed. In conclusion, the present study revealed that individual changes in training distribution generated an increased VO_2max_ in four out of six already well-trained cross-country skiers. Reduced total training volume (three out of six) and increased (four out of six) HIT volume were the most marked changes.

## Introduction

Maximal oxygen uptake (VO_2max_) is regarded as the most important single physiological factor for aerobic endurance performance in endurance sports, such as cross-country skiing (Ingjer, 1991; di Prampero, 2003; Sandbakk and Holmberg, 2017). Accordingly, strong relationships have been established between improved VO_2max_ and improved performance level in cross-country skiers (Ingjer, 1991; Mahood et al., 2001; Sandbakk et al., 2013; Johansen et al., 2020, 2021). In addition, more successful skiers have displayed higher VO_2max_ values compared with less successful skiers (Losnegard et al., 2013; Tønnessen et al., 2015; Sandbakk et al., 2016). Consequently, improvements of every individual skier’s VO_2max_ should be highly prioritized to improve performance level.

Cross-country skiers traditionally dedicate 80–90% of their endurance training at lower intensities (LIT), <82% of maximal heart rate (HR_max_) (Sandbakk and Holmberg, 2017). Therefore, less time have been spent in moderate- (MIT), 82–87% HR_max_, and high-intensity training (HIT), >87% HR_max_, throughout the preparation period (Losnegard et al., 2013; Sandbakk et al., 2016; Johansen et al., 2020). HIT has been shown to be an efficient way to improve VO_2max_ in both healthy moderately trained individuals (Helgerud et al., 2007; Støren et al., 2017) and highly trained endurance athletes (Støren et al., 2012; Sandbakk et al., 2013; Rønnestad et al., 2014, 2016). However, higher amount of HIT has been criticized for being too demanding and hamper physiological and performance adaptations in elite endurance athletes (Esteve-Lanao et al., 2007; Seiler, 2010; Slivka et al., 2010; Svendsen et al., 2016). In contrast, there have been few indications of a positive relationship between LIT and improved VO_2max_. However, LIT may serve as important to improve other performance-determining factors, i.e., technique-specific work economy (Haugnes et al., 2019).

Few studies have observed a significant improvement of aerobic capacity without considerable changes in training characteristics (i.e., training intensity distribution). A recent study by Johansen et al. (2020) revealed no improvements in aerobic capacity in well-trained national-level cross-country skiers within 6 months of season preparation (May to October). No to minor changes in training intensity distribution and time in different intensity zones were observed in that study. Additionally, few studies have followed well-trained cross-country skiers with initially no to minor improvements in aerobic capacity after a high LIT–low HIT program, to then change training characteristics considerably and study the subsequent physiological adaptations. To our knowledge, only Gaskill et al. (1999) did show positive adaptations of VO_2max_ and performance in a group of cross-country skiers after a year with higher amounts of HIT and maintained training volume, after no response was observed after a year with a high LIT–low HIT program. This is in accordance with the positive adaptations in both VO_2max_ and performance over shorter training periods in cyclists (Støren et al., 2012; Rønnestad et al., 2014) and cross-country skiers (Sandbakk et al., 2013; Rønnestad et al., 2016). However, there is still a need for more longitudinal investigations to better understand how to further develop VO_2max_ of national-level cross-country skiers.

Thus, the main purpose of this study was to evaluate changes in training distribution and subsequent effects on VO_2max_ among skiers who participated in the Johansen et al. (2020) study.

## Methods

### Study Design

The present study was a follow-up study to the “no change-no gain” study (Johansen et al., 2020). In Johansen et al. (2020), none of the participants changed their training habits substantially. As a follow-up, six of the skiers volunteered to participate motivated by self-induced changes in training including more HIT. The main purpose of the present study was thus to evaluate these changes in training and the concurrent effects on VO_2max_. After the termination of tests in October 2019 in the Johansen et al. (2020) study, the participants returned to the laboratory for a VO_2max_ test in February 2020 and halfway into a new preparation period in August 2020. All daily training was registered throughout the follow-up period.

### Subjects

Six national level cross-country skiers (three males and three females) participated in the present follow-up study, after previous participation in Johansen et al. (2020). In the Johansen et al. (2020) study, all included skiers were defined as well-trained cross-country skiers with VO_2max_ values ± 62 and ± 70 ml·kg^−1^·min^−1^ for females and males, respectively, well above gender means (Åstrand et al., 2003). Also, the skiers were labeled as well-trained based on competition results and training history (Johansen et al., 2020). The participants in the present study volunteered to continue their training registration and test participation of VO_2max_ after the termination of the study of Johansen et al. (2020). There were thus no separate inclusion criteria for participation in the present study. Subject characteristics for the six skiers are presented in Table 1. In accordance with the Declaration of Helsinki, all skiers gave their written informed consent to participate in the present study after receiving all necessary information about the study. The regional ethics committee of Southeast Norway (REC) evaluated and approved the follow-up study.

**TABLE 1:** Subject characteristics at February 2020.

TP	Gender	Age (years)	BW (kg)	ΔBW	Height (cm)
1	Female	32	76.5	+1.2	175
2	Female	24	61.4	+4.3	175
3	Male	17	72.2	+3.4	182
4	Female	26	69.1	+1.0	174
5	Male	31	75.9	+0.3	183
6	Male	25	71.1	−3.4	175
Mean ± SD (CV)	–	25.8 ± 5.4 (21.0)	71.0 ± 5.5 (7.7)	1.1 ± 2.7 (238.2)	177.3 ± 4.0 (2.3)

*Values are presented as individual values and mean ± standard deviation (SD) with coefficient of variance (CV) in parenthesis from the test in February 2020. *TP*, testperson; *BW*, body weight; Δ*BW*, change in body weight from summer 2019 to summer 2020; *kg*, kilogram; *Cm*, centimeters.*

### VO_2max_ Measurements

The VO_2max_ measurements were conducted on a Woodway PPS 55 sport treadmill (Waukesha, WI, United States), calibrated for speed and incline, while running. All VO_2_ measurements were taken by the metabolic test system, Cortex MetaLyzer II (Biophysics GmbH, Leipzig, Germany), with measurements every 10 s. According to the manufacturer’s instructions, the gas analyzers were calibrated with ambient air and certified calibration gases (16% O_2_ and 4% CO_2_) before each test. A 3-L calibration syringe (Biophysics GmbH, Leipzig, Germany) was used to calibrate the flow sensors. The heart rate (HR) of each participant was measured by Polar s610 HR monitors (Kempele, Finland) or by his or her own HR monitors.

The testing procedures of VO_2max_ measurements were similar to the protocols used in Johansen et al. (2020) and described in detail in Sunde et al. (2019). In brief, all participants were instructed to do only light training 24 h before attending to the laboratory. Prior to the VO_2max_ test, all participants were instructed to consume similar meals and drinks as prior to previous VO_2max_ tests in Johansen et al. (2020). Warm-up procedures were self-selected and lasted for at least 10 min. The starting intensity was set to an inclination of 6% and an 8–8.5 and 9–10 km·h^−1^ for females and males, respectively. During the first minute of the test, inclination was increased by 1% every 30 s until 8% was reached. From that point, only speed was increased by 0.5 km·h^−1^ every 30 s until voluntary exhaustion. To determine VO_2max_, the mean of the three highest consecutive VO_2_ measurements were used. To objectively evaluate if a true VO_2max_ was reached, a flattening of the VO_2_ curve, HR ≥ 97% of HR_max_, respiratory exchange ratio (RER) ≥1.05, and rate of perceived exertion (Borg scale 6–20) ≥17 was used as criteria.

Allometric scaling has in previous studies been shown to be important in order to compare VO_2max_ in athletes with different anthropometries and to compare individuals across sexes (Bergh and Forsberg, 1992). To compare the present results with previous results on cross-country skiers, allometric scaling with body weight raised to the power of 0.67 was used in addition to relative and absolute VO_2max_ values.

### Training Registration

All recruited skiers were instructed to train according to their own training programs and worked out by themselves and by their coaches throughout the whole study period. However, after the winter tests (February 2020), all participants voluntarily chose to change their training characteristics gradually until August 2020. Primarily, the skiers and their coaches planned these changes after their own wishes, while research personnel did only contribute to training discussions with the participants. Training performed from February 2019 to February 2020 (*P*_1_) represented the training performed during the study of Johansen et al. (2020). The next 6 months from February to August 2020 were divided into two periods; training from February to July (*P*_2_) and training from July to August (*P*_3_).

Training registration was performed similar to the procedures described in detail in Johansen et al. (2020). Briefly, the same research personnel registered and controlled every training session by use of training diaries throughout the whole study period. All endurance training were registered based on HR measurements and categorized into three separate HR intensity zones as “time in zone.” These three intensity zones were low-intensity training (LIT, <82% HR_max_), moderate-intensity training (MIT, 82–87% HR_max_), and high-intensity training (HIT, >87% HR_max_). The set HR zones used in the training registration were chosen to be below (LIT), around (MIT), and above LT (HIT) and corresponds to the intensity zones provided by the Norwegian Olympic Federation (Tønnessen et al., 2015). This training intensity categorization into three different zones has previously been used in several studies investigating training effects on VO_2max_ (Losnegard et al., 2013; Tønnessen et al., 2015; Sandbakk et al., 2016; Støren et al., 2017; Johansen et al., 2020, 2021). The HIT zone is also representative for the zone shown to be an effective training intensity to provoke VO_2max_ changes in already fit individuals (Helgerud et al., 2007; Støren et al., 2012; Bratland-Sanda et al., 2020).

The participants were asked to report if anything, included nutritional aspects, deviated from normal living during the study period. However, blood variables, such as iron or vitamin status, were not measured during the same period.

### Statistical Analyses

The data from the present study was presented and interpreted both as individual results from each participant and as mean ± standard deviation (SD) for results on a group level. QQ plots and normality tests (Shapiro-Wilk) were used to evaluate normal distribution in key variables. Despite a non-significant Shapiro-Wilk test, which indicates a normal distribution, the low sample size in the present study makes it challenging to evaluate whether the sample is normally distributed or not. Thus, both parametric and non-parametric statistics were used to evaluate changes in VO_2max_ over time (Paired sample *t*-test and Wilcoxon *t*-test). However, non-parametric tests did reveal almost identical *p*-values as parametric tests. Thus, parametric results are presented in the results. To evaluate differences in training, a GLM Univariate test was used.

The statistical package for social science version 26 (SPSS, IBM, Chicago, IL, United States) was used for all statistical analyses performed. A *p*-value <0.05 was taken as the level of significance in all two-tailed tests.

## Results

A total of 4,293 training sessions were registered and analyzed from February 2019 to August 2020 for all skiers combined, with an interindividual range of 6–12 training sessions per week^−1^ on average. None of the participants reported any long training breaks due to injuries or sickness during the study period. In addition, no periods of altitude training were reported. No deviations from their normal diets were reported, and body weight did not change significantly during the study period for any participants during the whole follow-up period (Table 1). Training characteristics for total training volume and HIT volume for the three training periods are presented in Table 2.

**TABLE 2:** Weekly training characteristics during the study period.

TP	*P*_1_ (February 2019–February 2020)			*P*_2_ (February 2020–July 2020)			*P*_3_ (July 2020–August 2020)		
			
	Total	HIT min	HIT%	Total	HIT min	HIT%	Total	HIT min	HIT%
1	508.6	13.8	2.7	469.5	17.8	3.8	396.1	52.4	13.2
2	668.2	40.0	6.0	911.9	27.6	3.0	1,068.1	105.3	9.9
3	903.7	48.1	5.3	1,026.7	52.6	5.1	986.4	83.9	8.5
4	738.9	55.8	7.6	916.5	38.2	4.2	1,228.5	73.5	6.0
5	891.7	58.5	6.5	623.1	52.9	8.5	859.8	59.3	6.9
6	866.1	22.8	2.6	1,016.8	15.9	1.6	1,215.2	14.5	1.2
Mean ± SD (CV)	762.9 ± 155.7 (20.4)	39.8 ± 18.1 (45.5)	5.1 ± 2.1 (41.2)	839.4 ± 206.7 (24.6)	35.2 ± 18.0 (51.1)	4.4 ± 2.3 (52.3)	959.0 ± 309.1 (32.2)	64.8 ± 31.0 (47.8)	7.6 ± 4.0 (52.6)

*Values are presented in minutes per week and percent, and mean ± standard deviation (SD), with coefficient of variance (CV) in percentage in parenthesis. *P*_1_, training period from February 2019 to February 2020; *P*_2_, training period from February 2020 to July 2020; *P*_3_, training period from July 2020 to August 2020; *TP*, test person; *Total*, total training volume; *HIT*, high-intensity aerobic training (>87% of HR_*max*_). Total training also includes strength training, LIT, MIT and other training. No significant differences.*

In general, training characteristics in *P*_1_ were representative for the training performed in Johansen et al. (2020) for all skiers. On a group level, no statistically significant differences were detected in either total training volume or HIT volume between the different training periods. This was found despite an average increase of 25.7 and 14.2% in total training volume, and a 62.8 and 84.1% increase in HIT volume in *P*_3_ compared with *P*_1_ and *P*_2_, respectively. The lack of significant changes in training distribution on a group level was due to large individual differences in training changes.

Mean VO_2max_ decreased slightly (−2.8%) but not significantly from August 2019 to February 2020. A significant increase in VO_2max_ (ml·kg^−1^·min^−1^) was observed from the mean 2019/2020 value to August 2020 (Table 3, *p* < 0.05).

**TABLE 3:** Development of VO_2max_ (ml·kg^−1^·min^−1^).

TP	August 2019	October 2019	February 2020	Mean 2019/2020	August 2020	ΔVO_2max_ (%)
1	64.1	62.3	61.3	62.6	68.3	+9.1
2	59.5	–	57.8	58.7	59.3	+1.0
3	70.8	71.2	69.0	70.3	77.5	+10.2
4	63.2	61.1	63.6	62.6	61.5	−1.8
5	68.9	69.5	63.6	67.3	72.2	+7.3
6	72.4	71.4	72.2	72.0	78.5	+9.0
Mean ± SD (CV)	66.5 ± 5.0 (7.5)	67.1 ± 5.0 (7.5)	64.6 ± 5.2 (8.0)	65.6 ± 5.1 (7.8)	69.6 ± 8.0* (11.5)	5.8 ± 5.0 (6.1)

Values are mean, and mean ± standard deviation (SD) with coefficient of variance (CV) in percentage in parenthesis. *TP*, test person; VO_2max_, maximal oxygen uptake; *ml·kg^−1^·min^−1^*, milliliters oxygen per kilogram bodyweight per minute; ΔVO_2max_, delta percentage change in VO_2max_ from mean 2019/2020 to August 2020. *p < 0.05 significantly different from mean VO_2max_ 2019/2020 and February 2020.

**FIGURE 1:**
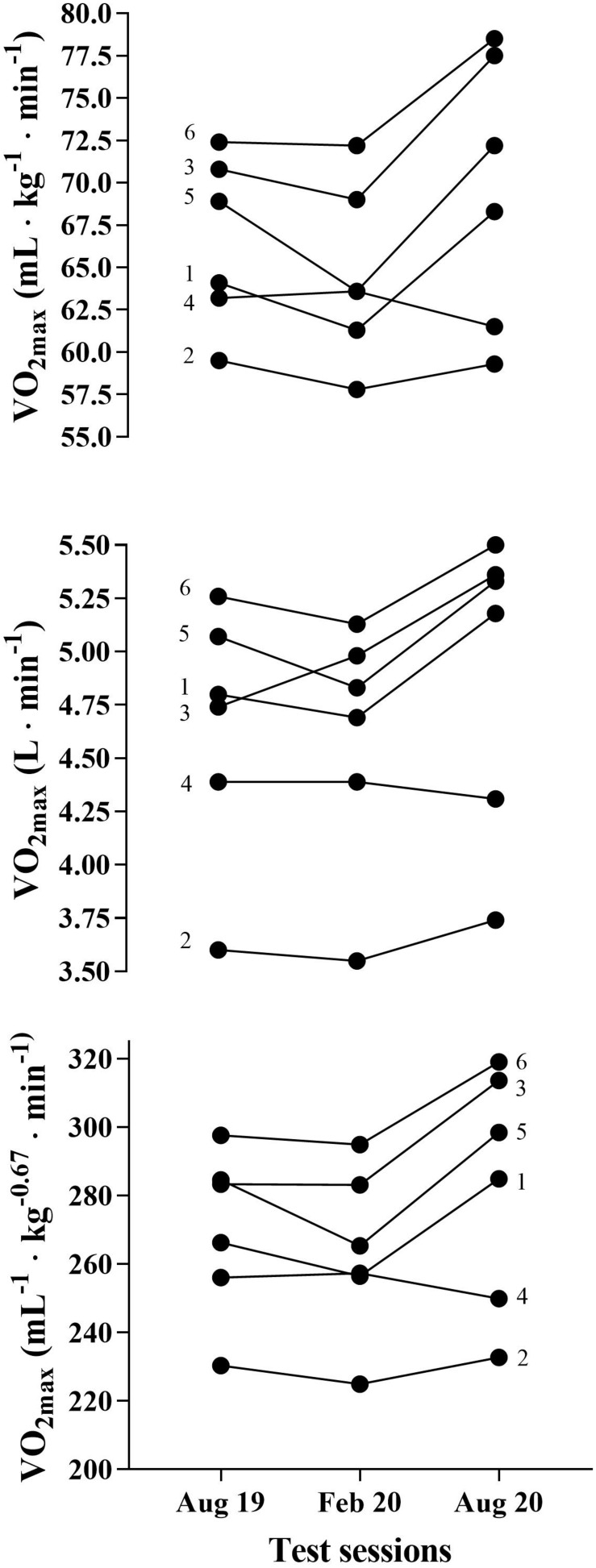
Development of VO_2max_ from August 2019 to August 2020. VO_2max_, maximal oxygen uptake; ml·kg^−1^·min^−1^, milliliters per kilogram bodyweight per minute; L·min^−1^; liters per minute; ml·kg^−0.67^·min^−1^, milliliters per kilogram bodyweight raised to the power of 0.67 per minute.

Four out of six skiers increased HIT volume (+59.5%–281.5%), one skier maintained HIT volume, and one skier slightly reduced HIT volume in *P*_3_. Changes from *P*_1_ to *P*_2_ were largely characterized by maintenance or reductions in HIT for all skiers. Four out of six skiers increased total training volume, while two reduced total training volume (Table 2) during the follow-up period (*P*_2_ + *P*_3_). VO_2max_ improved in four out of six skiers (7.9–10.2%), while one maintained and one decreased VO_2max_ (Table 3; Figure 1). Due to the individual responses in VO_2max_ after the performed changes in training distribution by the participants, no significant correlations were observed between delta VO_2max_ and delta total training volume or HIT volume.

## Discussion

The main findings of the present study were the individual changes in training intensity distribution and VO_2max_. The six skiers had previously showed no effect on VO_2max_ after maintaining high-volume LIT and low-volume HIT. Four of these skiers, as well as the mean of all skiers improved VO_2max_ after individual changes in training intensity distribution. The most markedly change in training was an increase in HIT volume by four of the six skiers. Three of the six skiers lowered their total training volume. The largest improvements in VO_2max_ were among those lowering the total training volume and/or increasing the amount of HIT. However, one skier also improved VO_2max_ after increased total training volume. The skiers who increased total training volume and the amount of HIT concurrently improved VO_2max_ the least.

Four of the skiers measured their highest VO_2max_-value after the follow-up period. This improvement was observed after an average increase of 60–85% in HIT volume the last month prior to the last VO_2max_-test (August 2020) compared with the preceding 17 months. The increase in HIT was accompanied by a decrease in total training volume, mainly LIT, in three of the skiers. The effectiveness of HIT in improving VO_2max_ is well documented in endurance athletes in both shorter periods (5–12 weeks, Sandbakk et al., 2013; Rønnestad et al., 2014, 2016) and longer periods (>12 weeks, Gaskill et al., 1999; Ingham et al., 2012; Støren et al., 2012; Solli et al., 2019). Accordingly, the improved aerobic capacity in the present skiers was not surprising. However, large individual differences in VO_2max_ response following different training changes were observed in the present study. Such individual differences have previously been observed after different training protocols with high HIT volumes (Bratland-Sanda et al., 2020) or traditional endurance training protocols (Gaskill et al., 1999).

Comparing the male skiers in the present study, almost similar VO_2max_ responses were displayed among all three skiers after considerable differences in training characteristics and training changes in *P*_2_ and *P*_3_ compared with *P*_1_. All male skiers trained equal amounts of training in *P*_1_. However, skier 6 trained almost 7 h·week^−1^ more compared with skier 5 in both *P*_2_ and *P*_3_. In contrast, skier 5 trained approximately 0.5 h·week^−1^ more HIT compared with skier 6. Thus, with almost two-thirds of the total training volume and almost three times higher volumes of HIT compared with skier 6, the two skiers displayed an almost similar improvement in VO_2max_ during the follow-up period. However, skier 3 increased his total training volume from *P*_1_ to *P*_2_, while HIT volume was maintained. In *P*_3_, total training volume was reduced and the HIT volume increased. Comparable results were observed for the females, where skier 1 displayed the lowest total training volume among all skiers, especially in *P*_3_. She steadily reduced her total training volume throughout the follow-up period, while she added 280% HIT in *P*_3_ compared with *P*_1_. Compared with the two other females (2 and 4), which increased both total training volume and HIT volume in *P*_2_ and *P*_3_, she trained ∼50 and ∼65% less in *P*_2_ and *P*_3_, respectively. In addition, despite a lower HIT volume in both *P*_2_ and *P*_3_ compared with the two other females, skier 1 was the only female skier in the present study who improved VO_2max_ considerably (+9.1%) vs. + 1.0% and −1.8% in skiers 2 and 4, respectively.

The results from skiers 1, 3, and 5 are in agreement to the case study of Støren et al. (2012), where a national-level cyclist displayed considerable enhancements in VO_2max_ and cycling performance after lower total training volumes and higher volumes of HIT. In addition, a similar training program generated positive adaptations in VO_2max_ and performance among American cross-country skiers (Gaskill et al., 1999). These studies thus suggest that athletes may benefit from lowering their total training volume and/or increase HIT volume for a certain period to develop their aerobic capacity. In addition, a lower total training volume may generate sufficient restitution and physiological and mental surplus between HIT sessions. Accordingly, Sandbakk and Holmberg (2017) has proposed that the quality of each HIT session may be as important as the amount of HIT in already well-trained cross-country skiers. However, in the present study, skier 6 actually increased VO_2max_ after an increase in total training volume, but not HIT. This underlines the large individual responses to the individual changes in training.

Several previous studies have reported increased HIT volume to hamper further development in both endurance performance and VO_2max_ (Slivka et al., 2010; Svendsen et al., 2016) and induce higher risk of overtraining syndrome (Seiler, 2010). However, in those same studies, both HIT and total training volume (higher volumes of LIT, MIT, and HIT) are increased considerably (Slivka et al., 2010; Svendsen et al., 2016). This leads to a large increase in total training load not only generated by the increased HIT volume. Accordingly, the skiers in the present study that showed no improvement in VO_2max_ (skiers 2 and 4) did increase both total training volume and HIT volume considerably. Thus, we may speculate that the increased training volume combined with the increased HIT volume may have led to some sort of over-reaching in these skiers, as previously indicated in Bratland-Sanda et al. (2020). In contrast, the four responding skiers did not increase both factors at the same time. We therefore speculate that increments in HIT volume should be performed at least without increased, or preferably reduced, total training volume to generate beneficial adaptations in VO_2max_ in already well-trained cross-country skiers.

Skier 6 differed considerably compared with the other skiers in the present study, with the highest amount of total training and lowest amount of HIT. He actually lowered his HIT volume in *P*_2_ and *P*_3_ compared with *P*_1_, while he displayed a 9.0% improvement in VO_2max_ after the follow-up period. This result is in contrast to previous findings of more HIT generating beneficial adaptations for overall aerobic capacity (Gaskill et al., 1999; Helgerud et al., 2007; Støren et al., 2012; Sandbakk et al., 2013; Rønnestad et al., 2016). However, it is worth mentioning that this male skier reported 10–15 min more HIT per week and the highest monthly amount if HIT throughout all training periods approximately 3 months before the last VO_2max_ test. This period was combined with a ∼30% reduction in total training volume compared with the mean in both *P*_2_ and *P*_3_, and one of the lowest monthly training volumes recorded in the whole 18 months study period. One might speculate that this intensive training period with reduced total training volume and increased HIT volume could have generated a beneficial effect for VO_2max_ in this skier, and that this has been maintained until the last VO_2max_ test. Such intensive short-duration training periods have previously proved effective for improvement of VO_2max_ in well-trained endurance athletes (Laursen et al., 2005; Rønnestad et al., 2014, 2016).

### Practical Implications, Strengths, and Limitations

The large individual differences in total training volume and HIT volume leading to comparable responses in VO_2max_ observed in the present study, highlights the need for highly individualized training protocols to provoke further adaptations in already well-trained endurance athletes. In addition, well-trained cross-country skiers experiencing no further improvements in aerobic capacity over longer periods may benefit from changes in training distribution large enough to generate physiological responses. These training changes could include increased HIT volume and/or reduced total training volume over a certain time period, but other changes could of course also prove beneficial. In the present skiers, the most common beneficial change in terms of improvements in VO_2max_ was an increased or maintained amount of HIT combined with a reduced amount of total training during the follow-up period. This change was apparent in three out of four skiers that also improved their VO_2max_ during the study period. However, as observed in the present skiers, well-trained skiers should be careful with training changes leading to concurrent increments in total training volume and HIT volume. Thus, the present results should be of great interest for already well-trained, but stagnated cross-country skiers and their coaches aiming for higher VO_2max_.

One limitation of the present study was that we were unable to measure the direct impact of the increased VO_2max_ and changes in training distribution on cross-country skiing performance the following competitive season, due to the COVID-19 pandemic. It would have been interesting to also investigate the effect of such training changes on other relevant performance-determining factors (e.g., work economy).

The sample size in the present study is too small for generalization purposes. However, the study is one of very few investigations observing individual changes in training in already well-trained endurance athletes, and the subsequent responses on VO_2max_. A strength of the present study is thus that it evaluates what high-level athletes actually choose to do. The main purpose of this study was to evaluate and observe the effects of self-motivated changes in training distribution. Although the training and changes in training in itself could be difficult to replicate, we argue that the principle of observing skiers doing self-induced changes in training and test, i.e., aerobic capacity may be easy to replicate. However, the results may not be directly replicated due to the freely chosen change in training distribution and individual response to the performed change.

Thus, the scope of future investigations should be the effect of similar training changes on other relevant performance determining variables (e.g., work economy), combined with the effects on performance and VO_2max_. In addition, larger cohorts of well-trained to elite endurance athletes should be emphasized in future investigations.

## Conclusion

The present follow-up study revealed that individual changes in training distribution generated an increased VO_2max_ in four out of six already well-trained cross-country skiers. Reduced total training volume in three out of six skiers and increased HIT volume in four out of six skiers were the most marked changes. However, training changes leading to an increased total training volume combined with an increased HIT volume seemed less beneficial, in two out of six already well-trained cross-country skiers.

## Data Availability Statement

The raw data supporting the conclusions of this article will be made available by the authors, without undue reservation.

## Ethics Statement

The studies involving human participants were reviewed and approved by the Regional Ethics Committee of Southeast Norway. Written informed consent to participate in this study was provided by the participants’ legal guardian/next of kin.

## Author Contributions

J-MJ, ØS, and JH participated significantly in the planning and design of this study. J-MJ, ØS, AS, and LG participated in data collection. J-MJ, ØS, JH, AS, and LG participated in the writing of the manuscript. All authors read and approved the manuscript.

## Conflict of Interest

The authors declare that the research was conducted in the absence of any commercial or financial relationships that could be construed as a potential conflict of interest.
